# Epstein-Barr virus in the cerebrospinal fluid of HIV-positive patients. Observational cross-sectional study

**DOI:** 10.1186/1471-2334-14-S4-O27

**Published:** 2014-05-29

**Authors:** Ruxandra V Moroti-Constantinescu, Ioana D Olaru, Adriana Hristea, Ana-Maria Petrescu, Victoria Aramă, Elisabeta O Benea, Raluca M Hrişcă, Adrian Streinu-Cercel, Dan Oțelea, Dragoş Florea

**Affiliations:** 1 Carol Davila University of Medicine and Pharmacy, Bucharest, Romania; 2 National Institute for Infectious Diseases “Prof. Dr. Matei Balş”, Bucharest, Romania

## 

Different viruses are detected in the cerebrospinal fluid (CSF) of HIV-positive patients, with controversial implications in central nervous system (CNS) impairment. Objectives: to evaluate the presence, frequency and associations of Epstein-Barr virus (EB) in the CSF in HIV-positive patients.

We retrospectively analyzed CSF samples from HIV-positive adult patients (≥18 years old), with or without CNS impairment, collected between Oct 2011 and Oct 2012 in a Romanian tertiary infectious diseases hospital.

We performed a multiplex PCR coupled with electrospray ionization – time-of-flight mass spectrometry (Abbott Molecular) which can simultaneously detect: herpesviruses (1-5 and 8), polyomaviruses, enteroviruses, adenoviruses and parvoviruses.

The patients were characterized based on the immunological and virological HIV status, neurological findings (including neuroimagery and CSF exam) and comorbidities.

A number of 55 patients were analyzed, with a mean age of 33.4 years (31.5 median) and a male:female ratio of 1.9:1. The CD4 count had a mean of 32 (75 median and IQR=225).

The most frequently detected virus was EB in 20/55 cases.

The EB-positive subgroup had a similar mean age of 33.5 years (median of 31.0) but a different male:female ratio, of 1.2:1. The CD4 count had a mean of 105.7 (59.5 median and IQR=156).

The following analyses refer to the EB-positive group: 17/20 patients had neurologic impairment. Imagery was performed in 15 cases and was normal in 5. CSF cellularity had a median of 2/cmm, IQR=10, CSF-glucose a median of 48.0 mg/dL, IQR=34 and CSF-protein a median of 55.5mg/dL, IQR=49.5.

In 8/20 cases EB was found as singular agent in the CSF and in 12/20 it was associated with other microorganisms: other herpesviruses (3 cases), JC virus (4 cases), *Mycobacterium tuberculosis* (2 cases), *Cryptococcus neoformans* (2 cases) and *Toxoplasma gondii* (one case).

The CSF-HIV viral load was available in 12/20 cases, being detectable in 10 cases.

Regarding the neurological events (totalizing the neurological signs/symptoms, CSF exam and imagery), EB was probably causal in 4/20 cases, possibly causal in another 4/20 cases and was a bystander in 12/20 (3 cases with no impairment and 9 cases in which the impairments were strongly attributable to another germ).

EB is the most frequent agent found in the CSF (40% of HIV-positive cases), mainly in women. It can be mono-detected but it especially appears in multiple associations. In more than half of the cases EB acts as an innocent bystander.

